# Rosiglitazone attenuates atherosclerosis and increases high-density lipoprotein function in atherosclerotic rabbits

**DOI:** 10.3892/ijmm.2015.2072

**Published:** 2015-01-19

**Authors:** CHEN LI, YAN TU, TING-RONG LIU, ZHI-GANG GUO, DI XIE, JIAN-KAI ZHONG, YONG-ZHEN FAN, WEN-YAN LAI

**Affiliations:** 1 Divisions of Cardiology, Nanfang Hospital, Southern Medical University, Guangzhou, Guangdong 510515, P.R. China; 2 Divisions of Nephrology, Nanfang Hospital, Southern Medical University, Guangzhou, Guangdong 510515, P.R. China

**Keywords:** rosiglitazone, atherosclerosis, high-density lipoprotein, adenosine triphosphate-binding cassette transporter A1, scavenger receptor class B type I

## Abstract

Rosiglitazone has been found to have anti-atherogenic effects and to increase serum high-density lipoprotein (HDL) cholesterol (HDL-C) levels. However, *in vivo* studies investigating the regulation of adenosine triphosphate-binding cassette transporter A1 (ABCA1) and scavenger receptor class B type I (SR-BI) by rosiglitazone are limited. Moreover, the effects of rosiglitazone on the function and levels of HDL are unclear. In the present study, we investigated the effects of rosiglitazone on HDL function and its mechanisms of action in atherosclerotic rabbits. Our results revealed that rosiglitazone induced a significant increase in serum HDL-C levels, paraoxonase 1 (PON1) activity, [^3^H]cholesterol efflux rates, and the expression of ABCA1 and SR-BI in hepatocytes and peritoneal macrophages. The expression of ABCA1 was also increased in aortic lesions. Rosiglitazone markedly reduced serum myeloperoxidase (MPO) activity, aortic intima-media thickness (IMT) and the percentage of plaque area in the aorta. It can thus be concluded that in atherosclerotic rabbits, rosigitazone increases the levels of HDL-C and hinders atherosclerosis. Thus, it improves HDL quality and function, as well as the HDL-induced cholesterol efflux, exerting anti-inflammatory and antioxidant effects.

## Introduction

Clinical and epidemiological studies have demonstrated an inverse correlation between high-density lipoprotein (HDL) cholesterol (HDL-C) and the incidence of coronary artery disease (CAD) (1). Studies have also indicated that the quality of HDL can also influence the risk of CAD (2,3), and that HDL function is more important than the HDL-C plasma concentration (4). It is considered that HDL protects against atherosclerosis in multiple ways, including both through reverse cholesterol transport (RCT) and non-cholesterol-dependent mechanisms (5).

RCT is a multistep process through which HDL mobilizes excess cellular cholesterol from arterial-wall lipid-laden macrophages (foam cells) to plasma lipid-poor apolipoproteins, which are then transported to the liver, where cholesterol is catabolized or excreted into bile. The transport process is mediated by several transmembrane transporters, including adenosine triphosphate binding cassette transporter A1 (ABCA1) and scavenger receptor class B type I (SR-BI) (6). ABCA1 is an ubiquitous protein expressed abundantly in the liver, macrophages, brain and other tissues. ABCA1 promotes the efflux of cellular phospholipids and cholesterol to lipid-free apolipoprotein A (apoA)-I and other apolipoproteins. This is further supported by data indicating that the functional interactions between apoA-I and ABCA1 are necessary for the initial lipidation of apoA-I (7). Further evidence indicates that ABCA1 plays a role in the liver and intestines in initiating HDL formation and the RCT process (8). ABCA1 overexpression has been shown to protect C57BL/6 mice from diet-induced atherosclerosis (9). Another primary transmembrane receptor, SR-BI, is also highly expressed in the liver, steroidogenic glands and other tissues and cells, including the brain, the intestines, macrophages, endothelial cells and astrocytes. In addition to mediating selective lipid uptake from lipoproteins to cells, SR-BI mediates the bidirectional movement of unesterified cholesterol between lipoproteins and cells (10). The hepatic overexpression of SR-BI has been shown to be associated with decreased plasma levels of HDL-C, increased HDL cholesteryl ester clearance, increased biliary cholesterol content and the transport of cholesterol from the liver to the bile (11,12). The transgene or adenovirus-mediated hepatic overexpression of SR-BI has been found to markedly reduce atherosclerosis in various murine models of the disease (10).

Peroxisome proliferator-activated receptor (PPAR)γ agonists, such as rosiglitazone, are extensively used in the treatment of type 2 diabetes (13). These agonists have also been shown to exert anti-atherogenic effects in subjects with or without diabetes (14–17). PPARγ is primarily found in adipose tissue and arterial wall cells, such as endothelial cells, vascular smooth muscle cells and monocytes/macrophages where it modulates lipid metabolism (18). Since SR-BI, ABCA1 and PPARs are all expressed in the liver, it is possible that the regulation of these proteins by PPARs may modulate the atheroprotective effects. Malerød *et al* (19) found that activated PPARγ increased hepatic SR-BI levels *in vitro*, which may lead to the increase in hepatic cholesterol uptake and the decrease in lipid accumulation in peripheral tissues. Furthermore, Llaverias *et al* (20) found that treatment with rosiglitazone significantly induced the mRNA and protein expression of ABCA1 and SR-BI and markedly reduced intracellular free cholesterol levels. However, to the best of our knowledge, few studies have evaluated the *in vivo* regulation of SR-BI and ABCA1 by PPARs, and the effects of PPARγ agonists on HDL quality remain unclear.

The benefit of rabbits as an animal model is that they express cholesteryl ester transfer protein (CETP). The objective of the present study was to investigate the effects of rosiglitazone on the expression levels of ABCA1 and SR-BI, as well as on the anti-atherosclerotic function of HDL in atherosclerotic rabbits. The rate of HDL-mediated RCT, the antioxidant properties of HDL and the pro-inflammatory state were also evaulated.

## Materials and methods

### Experimental animals

This study was carried out in strict accordance with the recommendations in the Guide for the Care and Use of Laboratory Animals of the National Institutes of Health. The experimental procedures were in accordance with guidelines set by the Animal Experiment Committee of Southern Medical University, Guangzhou, China. All animal care and procedures were approved by the Animal Experiment Committee of Southern Medical University. A total of 18 New Zealand white male rabbits (4 months old, weighing 2.0±0.1 kg) were provided by the Laboratory Animal Center of Southern Medical University. The rabbits were randomly divided into 3 groups (n=6 in each group) as follows: the control group, the atherosclerosis group and the rosiglitazone group. The animals in the control group, atherosclerosis group and rosiglitazone group were fed a regular diet, a high-fat diet supplemented with 1% (w/w) cholesterol, 8% lard (w/w) and 0.05% cholate (w/w) and a high-fat diet plus rosiglitazone (0.5 mg/kg body weight/day), respectively. The doses were based on those indicated in previous studies (21,22). Each rabbit consumed approximately 120 g of food daily. The rabbits were caged individually and had access to water *ad libitum* for 12 weeks, and were maintained under a 12-h day/night cycle. Fasting blood samples were collected via the auricular vein in tubes without anticoagulant to obtain serum. Following centrifugation (3,500 rpm, 15 min, 4°C), the blood samples were aliquoted and stored at −70°C until the biochemical measurements. Blood lipid analysis was performed at 0 and 12 weeks (at the end of the experiment). Other laboratory analyses were performed at the end of the study and all the experimental rabbits were sacrificed by an overdose of 25 mg/kg pentobarbital at the end of the 12-week experimental period, as previously described (23).

### Isolation of peritoneal macrophages and hepatocytes

At the end of the 12-week experimental period, the rabbits were anesthetized with 2% sodium pentobarbital. Under sterile conditions, the peritoneal macrophages were collected by peritoneal lavage with 200 ml phosphate buffer solution (PBS) and purified using the adherent method. Subsequently, using a modified method described by Zhao *et al* (23), the parenchymal hepatocytes were isolated by classic *in situ* two-step perfusion of the liver, with collagenase IV (0.05%) by enzyme digestion with collagenase II (2 mg/ml).

### Analysis of the HDL-mediated cholesterol efflux from peritoneal macrophages and hepatocytes

Experiments were performed as previously described (23,24) with minor modifications. Oxidized low-density lipoprotein (Ox-LDL) was obtained from human low-density lipoprotein (LDL), as previously described by Havel *et al* (25) and Pirillo *et al* (26). In this study, the concentration of Ox-LDL was 30 *μ*g/ml. Peritoneal macrophages and hepatocytes which were isolated as previously described, were planted at a density of 2×10^5^ cells/ml in 24-well culture dishes, and incubated with Dulbecco’s modified Eagle’s medium (DMEM)/F12 supplemented with 0.2% bovine serum albumin, 1 *μ*Ci/ml [^3^H] cholesterol (PerkinElmer Life Sciences, Inc., Boston, MA, USA) and 30 *μ*g/ml of Ox-LDL. Twenty-four hours later, to equilibrate cellular free cholesterol pools, the cells were washed once with serum-free medium, and then incubated for a further 12 h with DMEM/F12 supplemented with 0.3 mmol/l cAMP (Sigma, St. Louis, MO, USA). For free cholesterol efflux experiments, 10 *μ*g/ml apoA1 (Sigma) were added and the cells were incubated for 4 h. The incubation medium was collected and centrifuged before assessing the radioactivity using a counter. Cell monolayers were washed with PBS and lysed with 1 ml of 0.1 M NaOH. The radioactivity of the medium and cell lysates was measured by liquid scintillation spectrometry (Beckman Instruments, Inc., Fullerton, CA, USA). The cholesterol efflux was measured as the medium [^3^H]cholesterol radioactivity, representing a percentage of total [^3^H]cholesterol radioactivity (cells plus medium). Individual efflux values were calculated as averages of 3 determinations in each well.

### Measurement of ABCA1 and SR-B1 protein expression by flow cytometry

The measurement of the protein expression of ABCA1 and SR-B1 in the peritoneal macrophages and hepatocytes was performed as previously described in the study by Pirillo *et al* (27). Specifically the suspension of peritoneal macrophages and hepatocytes (2×10^5^ cells/ml) was mixed with either mouse anti-ABCA1 antibody (CB11308030) or mouse anti-SR-B1 antibody (CB41343199; Santa Cruz Biotechnology Inc., Santa Cruz, CA, USA) for 60 min at room temperature in test tubes. Each suspension was then washed twice with PBS and subsequently added to a labeled PE fluorescence antibody (sc-53749; Seretec Inc., Charlotte, NC, USA). Thirty minutes later, the cells were collected and subjected to fluorescence flow cytometry using a FACSCalibur and FACScan flow cytometer (Becton Dickinson, San Jose, CA, USA). The values were expressed by the ABCA1 and SR-B1 average protein contents per 100 detected cells.

### Measurement of ABCA1 and SR-B1 mRNA expression by reverse transcription quantitative (real-time)-polymerase chain reaction (RT-qPCR)

A total of 200 mg of liver tissue was powdered in liquid nitrogen. Total RNA was isolated from the rabbit livers using TRIzol reagent (Gibco-BRL, Gaithersburg, MD, USA). First-strand complementary deoxyribonucleic acid (cDNA) was synthesized with random primers and the First Strand cDNA Synthesis kit (Cat. no. C0210A; GeneCopoeia, Rockville, MD, USA). All primer sets were subjected to rigorous database searches to avoid potential conflicting transcript matches to pseudogenes or homologous domains within related genes. The sequences of the primers used for quantitative PCR (qPCR) were as previously described (28): ABCA1 forward, 5′-GAT GGC AAT CAT GGT CAA TGG-3′ and reverse, 5′-AGC TGG TAT TGT AGC ATG TTC CG-3′, yielding a 201-bp size product; SR-BI forward, 5′-CAG TGG GCA TTG TGT CCT GTC-3′ and reverse, 5′-GGC TCA GTG CAG GCT GAT GTC-3′, yielding a 286-bp size product; and GAPDH forward, 5′-GGA GCC AAA AGG GTC ATC-3′ and reverse, 5′-CCA GTG AGT TTC CCG TTC-3′, yielding a 346-bp size product. The qPCR chain reaction was carried out on a MX3000P thermocycler (Stratagene, La Jolla, CA, USA) and was used for detecting the products from the reverse-transcribed cDNA samples. The abundance of ABCA1 and SR-BI messenger ribonucleic acid (mRNA) was determined by SYBRI assay with GAPDH as the normalizer. The PCR reactions for each sample were performed in duplicate, and the relative gene expression was analyzed, as previously described (28).

### Quantification of aortic atherosclerosis by histological analysis and immunohistochemistry

After the animals were sacrificed, the entire aorta was removed and fixed in a 10% neutral buffered formaldehyde solution for 48 h. For the microscopic quantification of the lesion area, 3 segments were obtained from the aortic arch, the thoracic aorta and the abdominal aorta. All segments were embedded in paraffin, cut into 4-*μ*m-thick cross sections and stained with hematoxylin and eosin (H&E) for the histological examination. The percentage of plaque area, which was defined as the surface area of plaque/surface area of the whole intima, as well as the aortic intima-media thickness (IMT) were calculated. For the microscopic evaluation of ABCA1 and SR-B1 protein expression in the lesions of the aorta, immunohistochemistry was performed as previously described (29). Immunostaining for ABCA1 (Boster Biotechnology Co. Ltd., Wuhan, China) and SR-BI (Abcam Co. Ltd., Cambridge, MA, USA) was performed on paraffin-embedded aortic atherosclerotic sections using the specific antibody and a streptavidin-biotin peroxidase-complex (SABC). Antibody binding was visualized using SABC kits (Boster Biotechnology Co. Ltd.), diaminobenzidine (DAB) and 3-amino-9-ethylcarbazole (AEC) were used as the chromogen and Mayer’s hematoxylin as the nuclear counterstain. The sections were dehydrated, cleared, mounted and subjected to morphometric analysis. Images (H&E-stained and immunostained) were captured using an Olympus BX51 light microscope equipped with a DP70 digital camera (Olympus, Tokyo, Japan). Image Pro Plus 6.0 special image analysis software (Media Cybernetics Inc., Rockville, MD, USA) was used to quantify the images.

### Laboratory analyses

#### Serum lipid analysis

The serum triglyceride (TG), total cholesterol (TC), HDL-C and LDL cholesterol (LDL-C) concentrations were measured using an automated biochemical analyzer (Type AU5421; Olympus).

#### Assessment of serum paraoxonase (PON)1 activity

Serum PON1 activity was assayed according to the method described in the study by Beltowski *et al* (30), using the synthetic substrate phenyl acetate (Sigma). PON1 activity towards phenyl acetate was determined by measuring the initial rate of substrate hydrolysis within an assay mixture (3 ml) containing 2 mM phenyl acetate, 2 mM CaCl_2_ and 10 *μ*l of plasma in 100 mM Tris-HCl (pH 8.0). The absorbance was monitored for 90 sec at 270 nm and the enzymatic activity was calculated from the E_270_ of phenyl acetate (1,310/M/cm) and expressed in U/ml (where 1 U of arylesterase hydrolyzes 1 *μ*mol of phenyl acetate/min).

#### Assessment of serum myeloperoxidase (MPO) activity

MPO activity was determined using a MPO activity kit (Jiancheng Bioengineering Co, Nanjing, China) using commercially available reagents, according to the manufacturer’s instructions. Briefly, the serum samples were incubated in a 50 mM sodium phosphate buffer containing 1.5 M hydrogen peroxide and 0.167 mmol *o*-dianisidine dihydrochloride for 30 min. The increase in absorbance at 460 nm was recorded with the use of a spectrophotometer and the enzymatic activity was calculated from E_460_ = 11,300/M/cm. A unit of MPO activity is defined as the amount of enzyme degrading 1 *μ*mol H_2_O_2_ per minute at 37°C.

#### Statistical analysis

Data are presented as the means ± SEM. One-way ANOVA was used for analyzing differences in variables between groups at the same time point. When the value was P≤0.05, the least significant difference method was used for comparison. An independent sample t-test was used for analyzing the differences in variables between 2 groups at the same time point. Coefficients of correlation (r) were calculated by Pearson correlation analysis. SPSS 13.0 software was used for statistical analysis with a value of P<0.05 indicating a statistically significant difference.

## Results

### General animal characteristics

**Figure 1:**
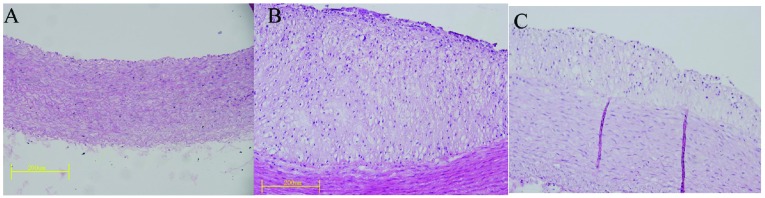
Representative micrographs of the intimal lesions. H&E staining was performed on aortic sections from rabbits in either (A) control group, (B) AS group and (C) rosiglitazone group. H&E, hematoxylin and eosin; AS, atherosclerosis.

There were no significant differences in serum lipid levels and body weight among the 3 groups at baseline (Table I). After 12 weeks of experiments, the atherosclerosis group had significant higher serum concentrations of TC, TG, HDL-C and LDL-C than the control group, while the rosiglitazone group had higher serum HDL-C concentrations and a slightly lower serum level of TC than the atherosclerosis group (Table I). There were no significant differences in body weight among the 3 groups throughout the experiment (Table I). Furthermore, mild to moderate atherosclerosis, characterized by the local thickening of the intima-media, was observed in the thoracic aortic wall of the rabbits fed a high cholesterol diet for 12 weeks (Fig. 1). The aortic IMT and the percentage of plaque area (surface area of plaque/surface area of whole intima) were significantly smaller in the rosiglitazone group compared with the atherosclerosis group (Table II). These results suggest that we successfully established a hyperlipidemic and atherosclerotic animal model.

### Rosiglitazone improves the HDL-induced cholesterol efflux in peritoneal macrophages and hepatocytes

**Figure 2:**
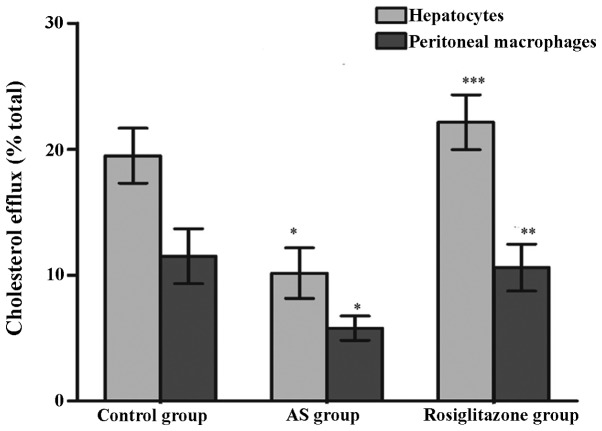
Comparison of HDL-induced cholesterol efflux rates from hepatocytes and peritoneal macrophages among the 3 groups. Data are presented as the means ± SEM, (n=6 in each group). ^*^P<0.01, vs. control group; ^**^P<0.01, ^***^P<0. 05, vs. AS group (ANOVA). HDL, high-density lipoprotein; AS, atherosclerosis.

We measured the rate of the HDL-induced cholesterol efflux in peritoneal macrophages and hepatocytes isolated from the 3 groups of rabbits and observed marked differences among the 3 groups. The cholesterol efflux rate in the peritoneal macrophages and hepatocytes from the rabbits in the atherosclerosis group was significantly lower than that in the control group rabbits (peritoneal macrophages: 16.48±4.10 vs. 24.93±3.85%, P<0.01; hepatocytes: 3.25±0.97 vs. 5.29±1.71%, P<0.05), while the peritoneal macrophages and hepatocytes from the rabbits treated with rosiglitazone showed a significantly enhanced HDL-induced cholesterol efflux as compared with the atherosclerosis group (peritoneal macrophages: 44.50±6.19 vs. 16.48±4.10%, P<0.01; hepatocytes: 8.50±1.18 vs. 3.25±0.97%, P<0.01; Fig. 2).

### Rosiglitazone increases ABCA1 and SR-B1 expression in peritoneal macrophages and hepatocytes

**Figure 3:**
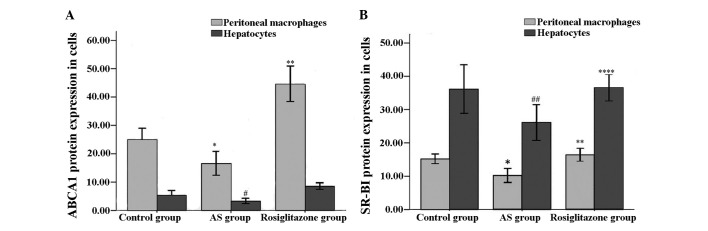
(A) ABCA1 protein expression in hepatocytes and peritoneal macrophages determined by flow cytometry. (B) SR-B1 protein expression in hepatocytes and peritoneal macrophages determined by flow cytometry. Data are presented as the means ± SEM, (n=6 in each group). ^#^P<0.05, ^*^P<0.01, ^##^P<0.001, vs. control group; ^**^P<0.01, ^***^P<0. 05, ^****^P<0. 001, vs. AS group (ANOVA). ABCA1, adenosine triphosphate binding cassette transporter A1; SR-B1, scavenger receptor class B type I; AS, atherosclerosis.

**Figure 4:**
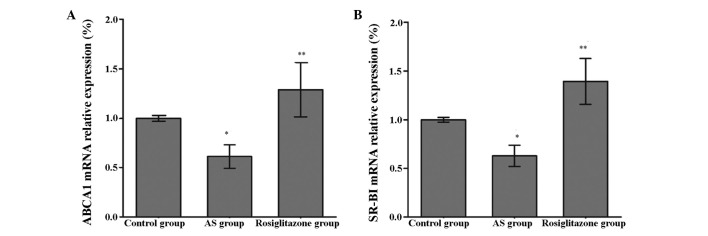
(A) Hepatocyte ABCA1 mRNA expression quantified by RT-qPCR. (B) Hepatocyte SR-B1 mRNA expression quantified by RT-qPCR. Data are presented as the means ± SEM, (n=6 in each group). ^*^P<0.01 vs. control group; ^**^P<0.01 vs. AS group (ANOVA). ABCA1, adenosine triphosphate binding cassette transporter A1; SR-B1, scavenger receptor class B type I; AS, atherosclerosis.

At the end of the 12-week experimental period, ABCA1 protein and mRNA expression in the peritoneal macrophages and hepatocytes was significantly lower in the atherosclerosis group compared with the control group (P<0.05; Figs. 3A and 4A). Compared with the atherosclerosis group, the rosiglitazone group had a significantly higher level of ABCA1 expression in the peritoneal macrophages and hepatocytes at both the mRNA and protein level (P<0.01; Figs. 3A and 4A).

Compared with the control groups rabbits, the atherosclerosis group showed a significant decrease in the peritoneal macrophage and hepatocyte expression of SR-B1 at both the mRNA and protein level (P<0.01; Figs. 3B and 4B). The mRNA and protein expression of SR-B1 in the peritoneal macrophages and hepatocytes increased significantly in the rosiglitazone group compared with the atherosclerosis group (P<0.01; Figs. 3B and 4B).

### ABCA1 and SR-B1 expression in atherosclerotic lesions

**Figure 5:**
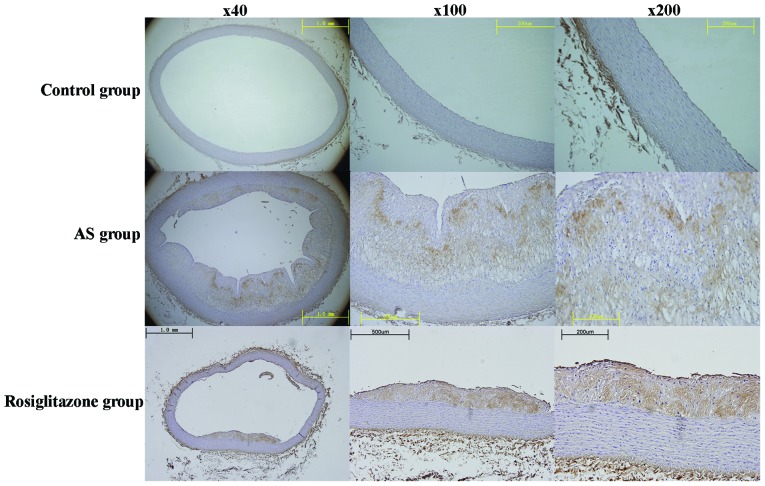
Aortic sections were subjected to immunohistochemical staining for ABCA1 protein localization. Representative images captured at magnification, ×40, ×100 and ×200. ABCA1, adenosine triphosphate binding cassette transporter A1; AS, atherosclerosis.

**Figure 6:**
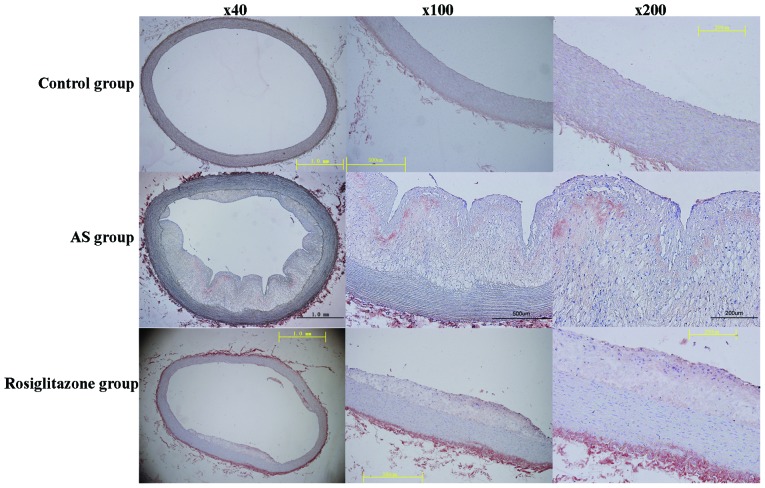
Aortic sections were subjected to immunohistochemical staining for SR-B1 protein localization. Representative images captured at magnification ×40, ×100 and ×200. SR-B1, scavenger receptor class B type I; AS, atherosclerosis.

Immunohistochemical staining revealed substantial ABCA1 and SR-B1 protein expression in the aortic plaques in both the atherosclerosis and rosiglitazone groups (Figs. 5 and 6). However, plaques and immunohistochemical staining in the aortic walls for ABCA1 and SR-B1 were negative in the control group, and no lesions were present. We further quantified ABCA1 and SR-B1 protein expression (by the percentage of positively stained areas and the staining intensity in the lesions by immunohistochemical staining) using Image Pro Plus 6.0 special image analysis software (Media Cybernetics Inc.). We found that ABCA1 protein expression was significantly increased in the rosiglitazone group, compared with the atherosclerosis group (Table III). However, there was no significant difference in SR-B1 protein expression in the aortic plaques between the 2 groups (Fig. 6).

### Rosiglitazone enhances HDL-associated antioxidant enzyme PON1 activity and suppresses oxidation enzyme MPO activity

**Figure 7:**
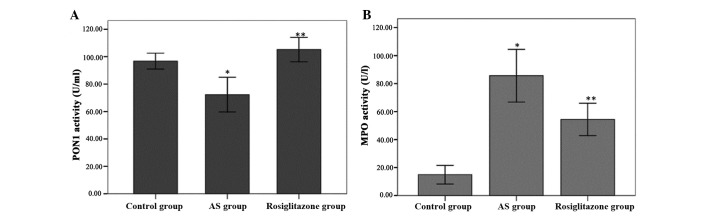
Treatment with rosiglitazone increased PON1 activity (U/ml) and reduced MPO activity (U/l) in the rosiglitazone group, compared with the AS group. (A) Serum PON1 activity. (B) Serum MPO activity. Data are presented as mean ± SEM, (n=6 in each group). ANOVA, P<0.05; ^*^P<0.01 vs. control group; ^**^P<0.05 vs. AS group. PON1, paraoxonase 1; MPO, myeloperoxidase; AS, atherosclerosis.

Serum PON1 activity (Fig. 7A) toward phenyl acetate was significantly inhibited in the atherosclerosis group compared with the control group (72.26±12.03 vs. 96.77±5.58 U/ml, P<0.001). In accordance with the results of a previous study (21), serum PON1 activity was markedly elevated in the rosiglitazone group compared with the atherosclerosis group (105.18±8.49 vs. 72.26±12.03 U/ml, P<0.01).

Serum MPO activity (Fig. 7B) was significantly higher in the atherosclerosis group compared with the control group (85.67±17.92 vs. 14.94±6.36 U/l, P<0.001). However, MPO activity was significantly attenuated in the rosiglitazone group compared with the atherosclerosis group (54.45±10.99 vs. 85.67±17.92 U/l, P<0.05).

Pearson correlation analysis was used to calculate the coefficients of correlation. We found no correlation between serum HDL-C levels and cellular cholesterol efflux, serum PON1 activity, serum MPO activity or IMT. However, the cellular cholesterol efflux in the peritoneal macrophages and hepatocytes, as induced by HDL, correlated with the protein expression level of ABCA1 and SR-B1 (data not shown) (in peritoneal macrophages, r=0.701, P=0.001; r=0.786, P<0.001, respectively; and in hepatocytes, r=0.763, P<0.001; r=0.813, P<0.001; respectively). In addition, IMT negatively correlated with serum PON1 levels and cellular cholesterol efflux in the peritoneal macrophages and hepatocytes (r=−0.675, P=0.002; r=−0.69, P=0.002; r=−0.816, P<0.001; respectively) and positively correlated with serum MPO activity (r=0.774, P<0.001) (data not shown).

## Discussion

This study demonstrates that rosiglitazone, an anti-diabetic medication with potential anti-atherogenic activity, attenuates atherosclerosis, increases serum HDL-C levels and improves the anti-atherogenic functions of HDL in atherosclerotic rabbits. Treatment with rosiglitazone for 12 weeks significantly decreased serum MPO activity and increased serum PON1 activity in this model. This indicates that rosiglitazone improves the HDL antioxidant and anti-inflammatory status. Moreover, we found that the administration of rosiglitazone improved the rate of the HDL-induced cholesterol efflux in peritoneal macrophages and hepatocytes, which was due to the upregulated expression of ABCA1 and SR-B1 at both the mRNA and protein level. New Zealand white rabbits were used as their lipoprotein profiles and lipid metabolism patterns are similar to those of humans, with differences in apoA-II and hepatic lipase levels (31).

Atherosclerosis is a chronic inflammatory disease that is initiated, in part, by the presence of Ox-LDL in the artery wall (32). Rosiglitazone is an orally active anti-diabetic drug in the thiazolidinedione drug class. It functions by binding as an agonist to the PPARγ receptor, where it inhibits the progression of atherosclerosis in patients (16). However, its anti-atherosclerotic mechanisms are not yet well understood. Since multiple epidemiological studies have established a low level of HDL-C as an independent risk factor for CAD (1), HDL has been under vigorous investigation as a therapeutic target for atherosclerosis. It has also been reported that PPARγ agonists increase HDL-C levels by 5 to 15% (33). However, there are conflicting reports on the association between HDL-C and atherosclerosis. Many data indicate that HDL-C levels and atherosclerosis are not correlated, and it has been suggested that the levels are associated with an increased risk, while others suggest a reduced risk of atherosclerosis. For example, it has been reported that the natural apo-A1 Milano mutation leads to low HDL-C levels, but does not confer an increased risk of cardiovascular events (34). Studies supporting HDL-C as a protective factor, contribute its effects to being mediated through multiple pathways, including RCT (particularly macrophage-specific RCT), anti-inflammatory, antioxidant, anti-aggregatory, anticoagulant and pro-fibrinolytic mechanisms (35). In addition, multifactorial actions, such as chronic inflammation and acute phase responses, can lead to the loss of normal HDL biological functions, resulting in dysfunctional HDL (4). Dysfunctional HDL exhibits chameleon-like properties that can protect arteries or enhance atherogenesis. For example, HDL isolated from some patients with CAD has been found to be ineffective as an antioxidant and, paradoxically appears to be pro-oxidant, as assessed by its lipid peroxide content (36). Given this complexity, it is not surprising that plasma HDL-C levels in a single assay do not necessarily correlate with HDL functions. Therefore, the evaluation of HDL function is more important than the quantification of its levels when assessing its atheroprotective properties. In this study, we investigated the effects of rosiglitazone on the antiatherogenic function of HDL in cholesterol-fed rabbits in order to obtain a better understanding of the potential antiatherogenic mechanisms.

RCT mediates the transport of cholesterol from peripheral cells back to the liver for excretion and is the most important antiatherogenic function of HDL. In this process, HDL mobilizes excess cellular cholesterol from arterial-wall macrophages to lipid poor plasma apolipoproteins, a transportation process primarily mediated by ABCA1 and SR-B1. It has been reported that the overexpression of ABCA1 increases the cholesterol efflux from cells (37). ABCA1-deficient mice have been shown to have reduced cholesterol efflux from macrophages to feces *in vivo* (38). The hepatic overexpression of SR-BI has beens shown to markedly reduce plasma HDL-C levels (39,40) and reduce atherosclerosis (41) in mice. Conversely, the gene deletion or attenuation of SR-BI in mice has been shown to result in substantially increased HDL-C levels (41,42), but markedly increased atherosclerosis (43). Additionally, Zhang *et al* (44) demonstrated that the modulation of hepatic SR-BI overexpression directly upregulates the rate of macrophage RCT *in vivo*. Previous studies have demonstrated that PPARγ agonists induce the expression of liver X receptor α (LXRα) and thereby stimulate ABCA1-dependent cholesterol efflux to apoA-1 or increase cholesterol efflux to HDL in an ABCG1-dependent manner (45–47). Consistent with previous findings, we found that rosiglitazone increased ABCA1 expression in peritoneal macrophages and hepatocytes at both the mRNA and protein level. We also provide the first demonstration, to the best of our knowledge, that ABCA1 increases the mRNA and protein expression of SR-B1 and enhances the HDL-induced cholesterol efflux in peritoneal macrophages and hepatocytes. Moreover, we observed ABCA1 and SR-B1 protein in aortic lesions by immunohistochemistry staining. We found that treatment with rosiglitazone was associated with increased ABCA1 protein expression in aortic lesions; however, there were no significant changes in SR-B1 expression. Furthermore, statistical analysis indicated that the cellular cholesterol efflux in peritoneal macrophages and hepatocytes was significantly positively correlated with the protein expression level of ABCA1 and SR-B1. Our data suggest that rosiglitazone improves cellular cholesterol efflux by upregulating ABCA1 and SR-B1 expression in cells.

In addition, it has been reported that HDL is an antioxidant and significantly reduces the oxidative modification of LDL. PON1 is an enzyme associated with the antioxidant properties of HDL (48). It has been reported that human PON protects LDL against oxidative stress, which helps explain its antiatherogenic mechanisms (49). A previous study provided direct evidence of a mechanistic link between the genetic regulation of PON and the resultant systemic oxidative stress (50). In our study, serum PON1 activity increased significantly following treatment with rosiglitazone, which is consistent with previous studies.

MPO, which is secreted by activated phagocytes, is one of the pivotal factors involved in the initiation of lipid oxidation of LDL (51). It has been demonstrated that MPO interacts with apoA1 and impairs cellular cholesterol efflux through ABCA1, leading to the formation of pro-inflammatory HDL. Thus, MPO inhibition may also be therapeutically valuable (52). It has been demonstrated that PPARγ agonists strongly regulate MPO gene expression through the Alu element encoding 4 hexamer repeats. In this study, we also found that rosiglitazone reduced serum MPO activity.

In conclusion, our *in vivo* study using rabbits demonstrated that treatment with rosiglitazone increased serum HDL-C levels, and improved the HDL-induced cholesterol efflux in hepatic cells and macrophages by upregulating ABCA1 and SR-B1 mRNA and protein expression in an atherosclerotic rabbit model. The anti-inflammatory and antioxidant effects of HDL may be promoted by decreasing serum MPO activity and increasing PON1 activity, thus deterring the development of atherosclerosis. These factors may contribute to the anti-atherogenic potential of rosiglitazone.

## Figures and Tables

**Table I:** Serum lipid and body weight profiles in the control, atherosclerosis and rosiglitazone groups.

Group	TC	TG	LDL-C	HDL-C	Body weight
Control					
0 weeks	1.85±0.12	1.04±0.19	1.00±0.20	0.47±0.12	2.01±0.08
12 weeks	1.79±0.21	1.02±0.15	1.09±0.23	0.45±0.12	2.77±0.04
AS					
0 weeks	1.77±0.15	1.10±0.14	1.00±0.20	0.48±0.12	2.07±0.06
12 weeks	23.26±3.30^a^	1.58±0.25^a^	18.09±4.04^a^	1.11±0.09^a^	2.88±0.08
Rosiglitazone					
0 weeks	1.78±0.12	1.02±0.14	0.98±0.14	0.47±0.07	2.04±0.02
12 weeks	19.78±1.68	1.54±0.15	15.57±1.81	1.94±0.30^b^	2.78±0.12

The serum levels of total cholesterol (TC), triglycerides (TG), low-density lipoprotein (LDL) and high-density lipoprotein (HDL) were measured in the rabbits in the control, atherosclerosis (AS) and rosiglitazone group. The units for TC, TG, LDL-C, HDL are in mmol/l and body weight are in kg. Data are expressed as the means ± SEM, n=6.

a P<0.001 vs. control

b P<0.05 vs. AS group.

**Table II:** Quantification of aortic atherosclerotic lesions in the control, atherosclerotic and rosiglitazone groups at 12 weeks.

Group	IMT (*μ*m)	Percentage of plaque area (%)
Control	203.21±30.61	
AS	527.42±85.16^a^	27.78±12.00
Rosiglitazone	291.46±50.18^b^	5.88±3.31^c^

The aortic intima-media thickness (IMT) and the percentage of plaque area (surface area of plaque/surface area of whole intima) were measured in the rabbits in the control, atherosclerotic (AS) and rosiglitazone groups. Data are expressed as the means ± SEM, n=6. IMT in the 3 groups:

a P<0.001 vs. control;

b P<0.001 vs. AS group (ANOVA). Percentage of plaque area of 2 groups:

c P<0.01 vs. AS group.

**Table III:** Quantification of immunohistochemical staining for ABCA1 expression in aortic atherosclerotic lesions among the control, atherosclerotic and rosiglitazone groups at 12 weeks.

Group	Staining intensity	Percentage of ABCA1 protein positive area (%)
AS	0.17±0.03	24.13±9.85
Rosiglitazone	0.22±0.03^a^	47.06±4.93^b^

The staining intensity and percentage of adenosine triphosphate-binding cassette transporter A1 (ABCA1) protein positive area in aortic plaques were measured between the rabbits in the atherosclerosis (AS) and rosiglitazone groups. Data are expressed as the means ± SEM, n=6.

a P<0.05

b P<0.001 vs. AS group.
